# A Diagnostic Masquerader in a Tuberculosis-Endemic Region: A Case of an Isolated Complicated Pulmonary Hydatid Cyst in an Adolescent

**DOI:** 10.7759/cureus.102920

**Published:** 2026-02-03

**Authors:** Shivangi Sinha, Harshit Khandelwal, Sabavath Arun, Amber Kumar, Shikha Malik

**Affiliations:** 1 Pediatrics, All India Institute of Medical Sciences, Bhopal, Bhopal, IND

**Keywords:** echinococcus granulosus (e. granulosus), pediatric infectious disease, pulmonary hydatid cyst, recurrent hemoptysis, tuberculosis mimic

## Abstract

Pulmonary hydatid disease, caused by *Echinococcus granulosus*, remains an underrecognized etiology of chronic respiratory symptoms in endemic regions, particularly when classical exposure history is absent. We describe the case of a 15-year-old adolescent girl from rural India who presented with a one-year history of progressive cough and intermittent hemoptysis, refractory to empirical antibiotic therapy. Imaging revealed a well-defined cystic lesion in the left upper lobe, and contrast-enhanced computed tomography (CECT) demonstrated the coexistence of crescent and water-lily signs, indicating a complicated pulmonary hydatid cyst with impending rupture. Serological testing for *Echinococcus granulosus* IgG was positive, while extensive evaluation for tuberculosis and fungal infections was negative, and no hepatic or extrapulmonary cysts were identified. The patient was treated with preoperative albendazole followed by successful lung-preserving surgical enucleation, resulting in complete clinical and radiological recovery without recurrence. This case underscores the diagnostic value of characteristic imaging findings and highlights the necessity of considering pulmonary hydatid disease in adolescents with persistent respiratory symptoms in tuberculosis-endemic settings, even in the absence of identifiable zoonotic exposure.

## Introduction

Hydatid disease is a zoonotic disease caused by the larval form of *Echinococcus granulosus* [1]. The life cycle of *Echinococcus *includes dogs as definitive hosts and livestock such as goats, sheep, camels, and pigs as intermediate hosts. Humans remain an accidental dead-end host and do not contribute to the completion of the life cycle. Although direct contact with definitive hosts or intermediate hosts is a known risk factor, transmission can occur indirectly through ingestion of contaminated water, food, or soil, even in the absence of direct contact with livestock or pets, thus accounting for a significant proportion of cases [2,3]. The disease continues to impose a substantial health burden in endemic regions, particularly in areas with poor sanitation and proximity between humans, dogs, and livestock [4]. Once ingested, larvae penetrate the intestinal mucosa and disseminate hematogenously, most commonly forming cysts in the liver and lungs. The liver remains the most commonly involved organ, followed by the lungs [3,5]. While hepatic cysts predominate in adults, pulmonary involvement is more frequent in children and adolescents, owing to greater pulmonary blood flow and increased elasticity of lung parenchyma [3]. Pediatric pulmonary hydatid cysts are often large, may remain asymptomatic for prolonged periods, and may occur as isolated lesions without hepatic involvement, making diagnosis challenging. Complications such as cyst rupture or secondary infection further obscure the clinical picture and may precipitate acute symptoms.

In tuberculosis-endemic regions, pulmonary hydatid disease is a well-recognized diagnostic masquerader. Chronic cough, hemoptysis, and persistent radiological opacities frequently overlap with pulmonary tuberculosis, lung abscesses, and fungal infections, often leading to empirical antimicrobial or anti-tubercular therapy and delayed diagnosis. Recognition of characteristic imaging signs is therefore critical, particularly in pediatric patients who do not respond to standard treatment and lack a classical exposure history [5].

In this context, we report a case of a complicated isolated pulmonary hydatid cyst in an adolescent girl, highlighting the diagnostic challenges encountered in a tuberculosis-endemic setting and underscoring the importance of maintaining a high index of suspicion for hydatid disease in children with chronic respiratory symptoms. 

## Case presentation

A 15-year-old female, the second born out of a non-consanguineous marriage, belonging to a lower socioeconomic status according to the Modified Kuppuswamy scale 2025 [6], and a resident of a rural background, presented with a one-year history of cough. A cough was present for the last year, which was insidious in onset, gradually progressive, and without any diurnal or postural variation. Initially, the cough was dry, but for the last month, it was associated with sputum production, which was mucoid in consistency and intermittently associated with blood-tinged, non-foul-smelling sputum. There was no history of fever, weight loss, night sweats, breathlessness, or tuberculosis contact. She had no direct contact with livestock or pets. No similar illnesses were reported in her family or community, and no history of travel was present. The patient was a resident of pucca house with adequate sanitation, including a separate kitchen and toilet.

The child was started on empirical outpatient antibiotic therapy, and no improvement in symptoms was noted. On presentation to our center, she was afebrile and did not show any signs of respiratory distress. Detailed respiratory examination showed bilaterally equal chest movement, with dullness noted on percussion over the left infraclavicular region. Breath sounds were reduced over the left mammary region. The findings were suggestive of an underlying fluid-filled lesion. The rest of the cardiovascular, abdominal, and neurological examinations were unremarkable.

**Figure 1:**
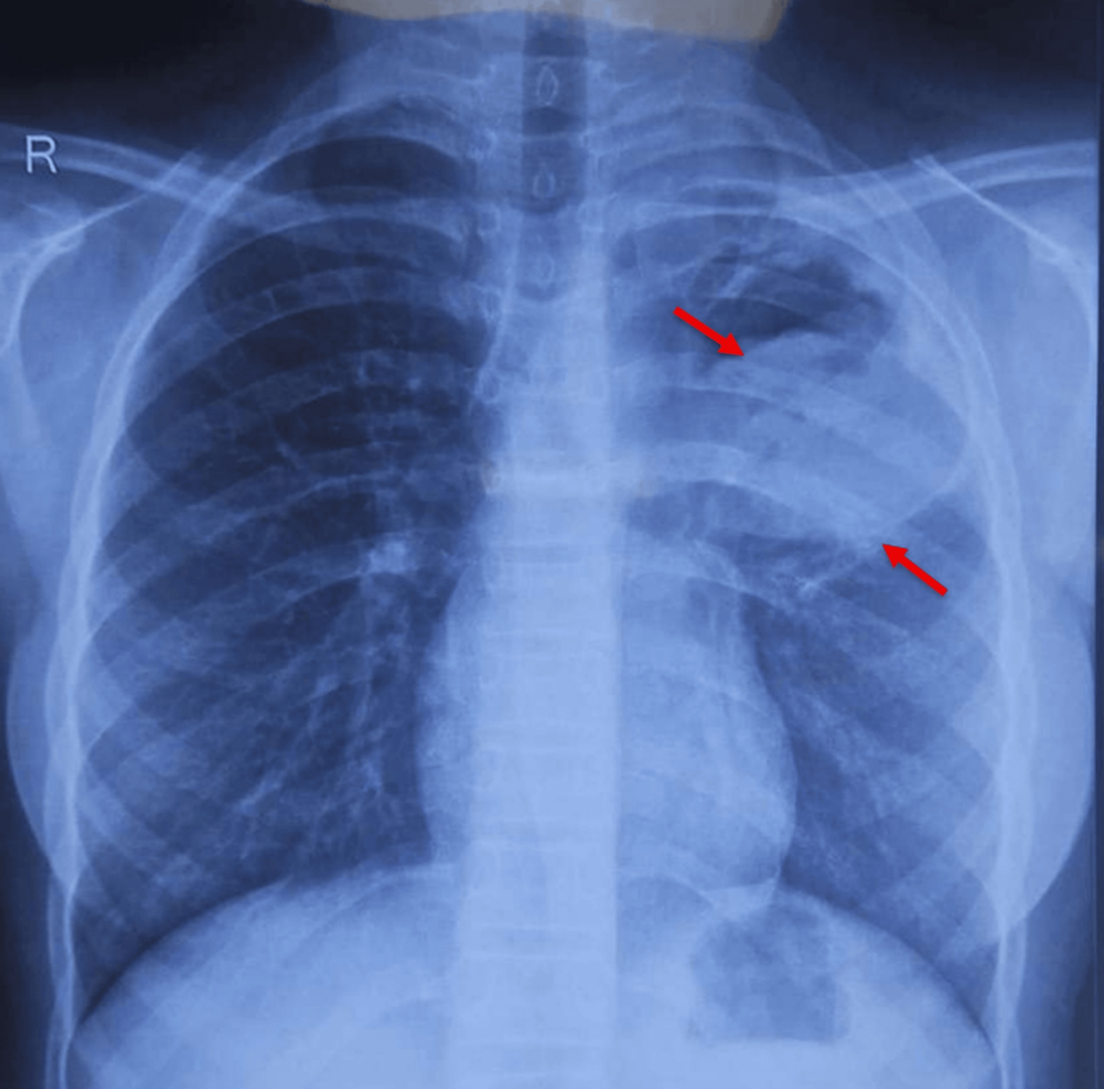
Chest radiograph (posteroanterior view) showing a well-defined round heterogenous opacity in the left upper lobe.

**Figure 2:**
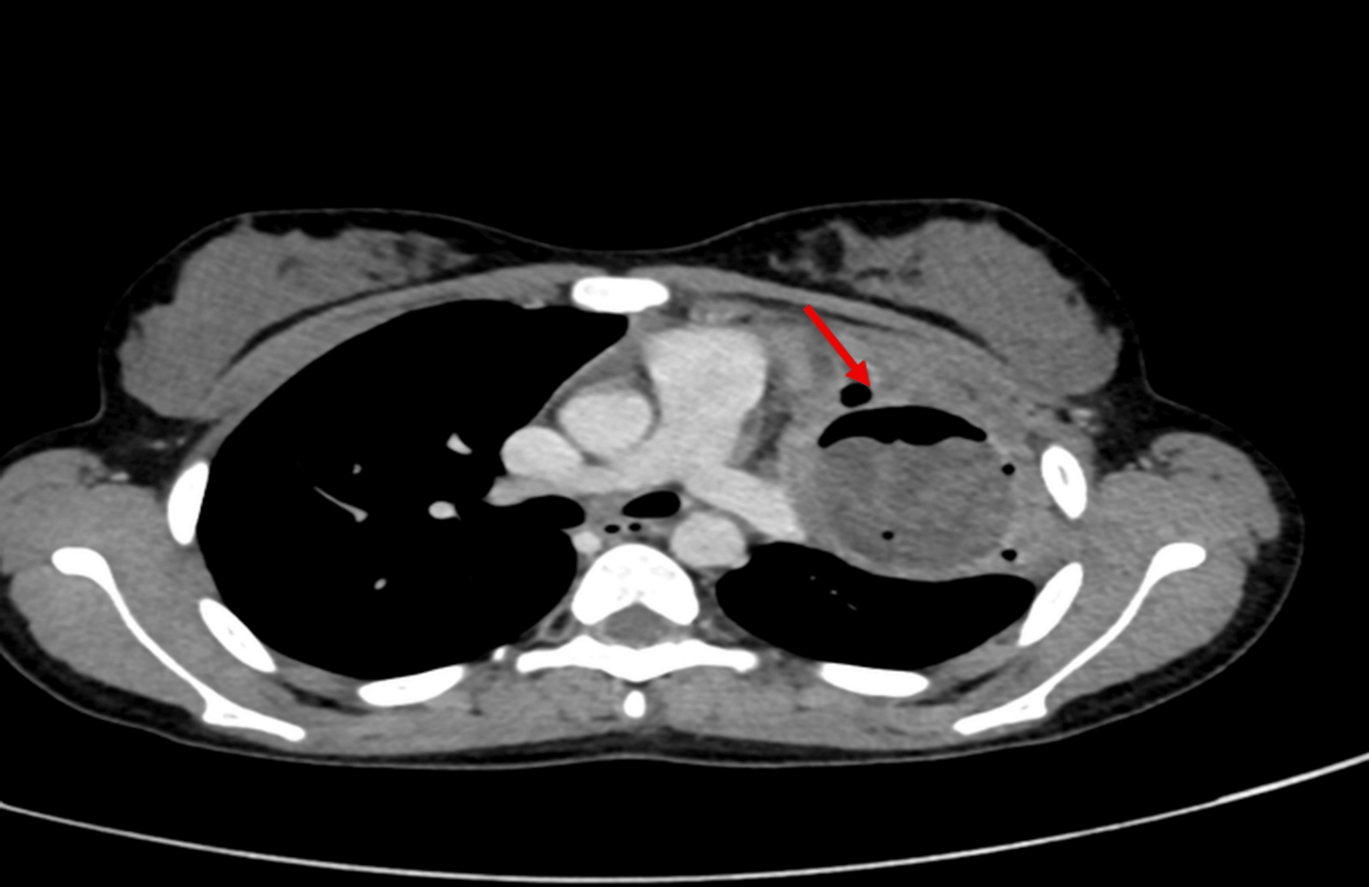
Contrast-enhanced computed tomography (CECT) of the thorax shows a crescent-shaped air pocket in the non-dependent portion of the lesion. There is a well-defined, thick-walled cystic lesion measuring approximately 5.5 x 5.6 x 5.9 cm (transverse x anteroposterior x craniocaudal) in the apical segment of the left upper lobe.

**Figure 3:**
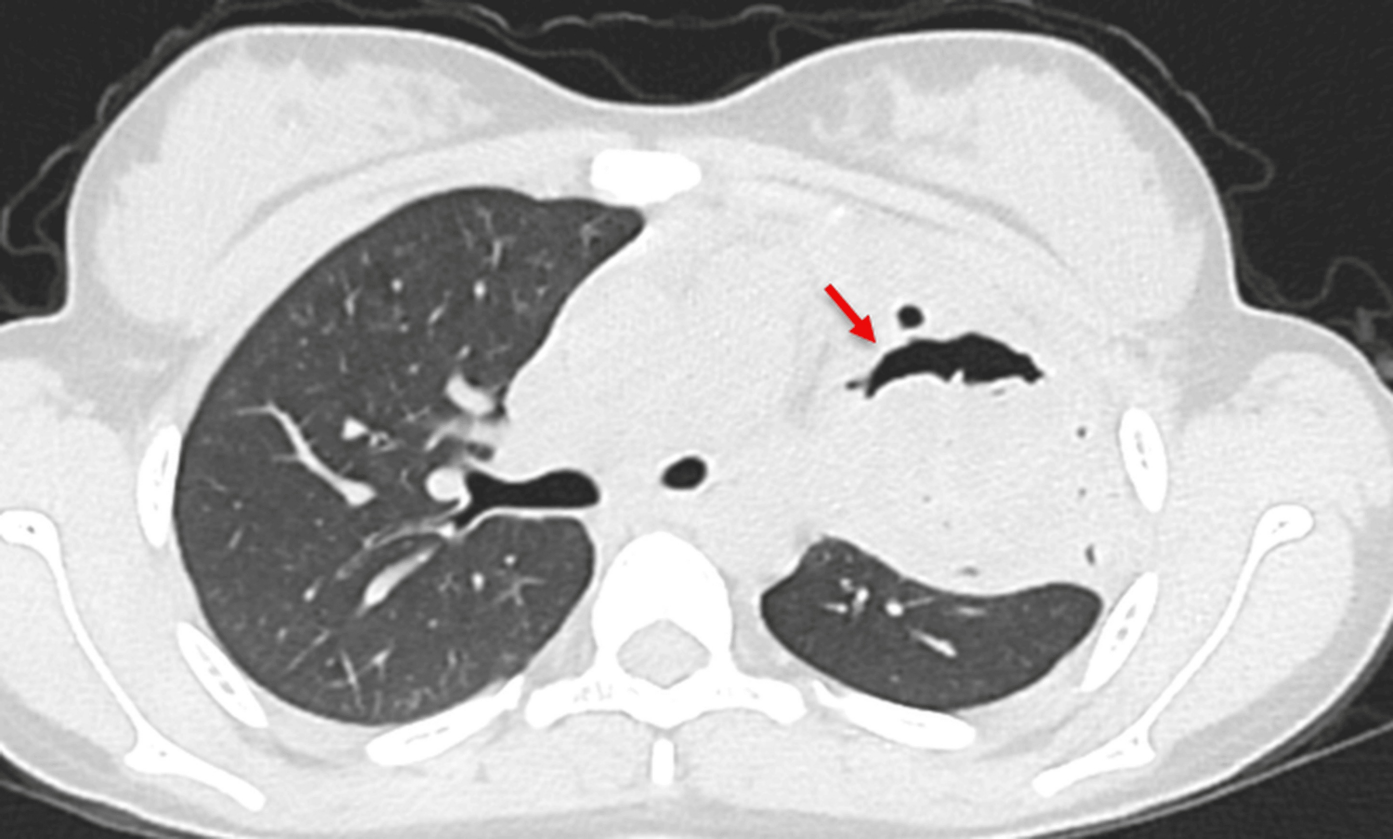
Contrast-enhanced computed tomography (CECT) of the thorax shows a crescent-shaped air pocket in the non-dependent portion of the lesion. A bleb of air dissecting into the cyst wall gives rise to the "signet ring sign," which may indicate imminent cyst rupture.

Initial laboratory investigations revealed a hemoglobin of 9.8 g/dL, with a peripheral smear showing normocytic, normochromic anemia as depicted in Table 1. White blood cell counts were within normal limits, and eosinophilia was not observed. A chest radiograph demonstrated a well-defined, round, heterogeneous, opaque lesion in the left upper lung, as depicted in Figure 1. Differential diagnosis of chronic inflammatory conditions, including tuberculosis, sarcoidosis, hydatid disease, lung abscess, bronchogenic cyst, and allergic bronchopulmonary aspergillosis, was considered, and the workup was planned. The workup for tuberculosis was negative. Contrast-enhanced computed tomography (CECT) of the thorax revealed a well-defined, thick-walled cystic lesion measuring approximately 5.5 x 5.6 x 5.9 cm (transverse x anteroposterior x craniocaudal) in the apical segment of the left upper lobe with peripheral rim enhancement, internal air foci, and a crescent-shaped air pocket (the “crescent sign”) consistent with impending rupture, as depicted in Figure 2 and Figure 3. Adjacent inflammatory changes were present, suggesting secondary infection. Abdominal ultrasonography was normal. Serological testing by IgG enzyme-linked immunosorbent assay (ELISA) was positive for *Echinococcus granulosus*.

**Table 1:** Result of laboratory investigations of the index case

Parameter	Patient Value	Unit	Reference Range
Hemoglobin	9.8	g/dL	11–15
Hematocrit	37.5	%	37–47
Mean corpuscular volume	89.7	fL	76–93
Mean corpuscular hemoglobin	28.4	pg	27–32
Mean corpuscular haemoglobin concentration	33.1	g/dL	32–36
White blood cell count	8.91	×10³/µL	4–11
Neutrophils	48.2	%	40 - 70
Lymphocytes	40.6	%	20 - 40
Eosinophils	1	%	0-2
Platelet count	304	×10³/µL	150–450
Total bilirubin	0.52	mg/dL	0.3–1.2
Direct bilirubin	0.12	mg/dL	<0.2
Aspartate aminotransferase	33.3	U/L	<35
Alanine transaminase	14.3	U/L	<35
Alkaline phosphatase	130.8	U/L	30–120
Total protein	7.9	g/dL	6.6–8.3
Albumin	4.3	g/dL	3.5–5.2
Urea	28.6	mg/dL	20–40
Creatinine	0.73	mg/dL	0.5–0.9
Sodium	135.8	mmol/L	136–145
Potassium	4.5	mmol/L	3.5–5.1
Chloride	100.4	mmol/L	98–107
Lactate dehydrogenase	423.2	U/L	<248
C-reactive protein	5.63	mg/L	<5.0
Antineutrophil cytoplasmic antibodies	Negative	-	
Total IgE	30.6	IU/mL	0–87
Hepatitis B surface antigen	Negative	-	
Anti-hepatitis C virus	Negative	-	
*Echinococcus *IgG	Positive	-	
Acid-fast bacilli test on bronchoalveolar lavage fluid	Negative	-	
Cartridge-based nucleic acid amplification test for *Mycobacterium tuberculosis*/rifampicin resistance	Not detected	-	
*Aspergillus *IgG/IgM	Negative	-	
Fungal culture	No growth	-	

The patient was started on oral albendazole at a dose of 400 mg twice daily for 21 days preoperatively, as the recommended minimal duration before surgery is 15 days. She subsequently underwent left posterolateral thoracotomy under general anesthesia. Intraoperative findings revealed a tense, partially ruptured hydatid cyst with internal membrane detachment and localized infection. The cyst was carefully enucleated without rupture or spillage, and the lung parenchyma was preserved. No pleural breach or bronchial fistula was identified.

Postoperatively, the patient remained stable, with no evidence of air leak, infection, or respiratory distress. She was discharged with albendazole therapy for an additional four weeks along with supportive therapy, including chest physiotherapy and dietary advice. At the three-month follow-up, she remained asymptomatic. Her hemoglobin levels had improved, and repeat chest imaging showed no evidence of recurrence.

## Discussion

Hydatid disease is a zoonotic infection caused by the larval stage of the *Echinococcus granulosus* worm. Over one million people are affected by hydatid cyst disease, thus representing a significant global health burden [4]. The disease is common in regions with livestock farming and limited public health services, namely parts of the Middle East, South America, Africa, and the Indian subcontinent. The most commonly involved organ remains the liver in adults, contrary to the lungs being most common in children [7-9].

Owing to more elastic lung tissues, pulmonary hydatid cysts often remain asymptomatic. The common symptoms reported in the literature consist of cough (61%), chest pain (44%), dyspnea (25%), sputum expectoration (17%), and hemoptysis (12%) [5]. The most common causes of hemoptysis include tuberculosis and bronchiectasis, whereas parasitic infections such as cystic echinococcosis are uncommon etiologies, as observed in the index case.

Most of the cases of pulmonary hydatid cysts follow an uncomplicated course of disease, while the remainder experience complications. A complicated hydatid cyst is defined as a cyst that has ruptured into the bronchus or pleural cavity, with or without infection [10].

Diagnosis of hydatid disease requires an intricate amalgamation of clinical, serological, and radiological features. Serological testing includes antibody testing by immunoassays and ELISA, with specificity and sensitivity ranging from 60%-90% and 90-98%, respectively [11]. Positive serological testing is seen in almost 65% of the patients with pulmonary hydatid cysts. Radiological features include a homogeneously appearing, round or oval cyst surrounded by normal lung parenchyma. Complicated hydatid cysts demonstrate a variety of characteristic imaging signs, including the crescent sign, water-lily sign, and double arch (Cumbo’s) sign. Additional features include the inverse crescent and signet ring signs, increased CT attenuation resembling a solid mass, thick-walled cavities, daughter cysts, the ring-within-a-ring appearance, mass-within-a-cavity (Monod’s) sign, and serpentine configurations such as the snake or whirl sign [11].

Medical management with albendazole is indicated both pre- and post-operatively to reduce cyst viability and the risk of recurrence. Surgical intervention remains the definitive treatment, especially for large or complicated cysts [3,12,13]. Lung-preserving surgical techniques, as employed in this case, minimize postoperative morbidity and preserve pulmonary function.

This case underscores the potential for pulmonary hydatid disease to remain undiagnosed for prolonged periods, particularly in tuberculosis-endemic regions where chronic cough and hemoptysis are often attributed to more common etiologies. Although there was no definite history of exposure to livestock or pets, pulmonary hydatid disease was suspected owing to characteristic radiological features. The co-existence of the crescent sign and water lily sign on CECT indicated partial cyst rupture with impending complications, emphasizing the importance of early cross-sectional imaging in children with persistent respiratory symptoms unresponsive to empirical antibiotics. Isolated pulmonary involvement without hepatic cysts, as seen in this patient, is uncommon but occurs more frequently in children and adolescents due to preferential pulmonary localization of the parasite [5,11].

## Conclusions

This case underscores the importance of including hydatid disease in the differential diagnosis of cystic pulmonary lesions in adolescents, even in the absence of direct livestock exposure. Timely recognition through imaging and serological evaluation, followed by appropriate medical and surgical management, can lead to excellent outcomes and prevent potentially fatal complications.
